# Embodied affectivity: on moving and being moved

**DOI:** 10.3389/fpsyg.2014.00508

**Published:** 2014-06-06

**Authors:** Thomas Fuchs, Sabine C. Koch

**Affiliations:** ^1^Phenomenological Psychopathology and Psychiatry, University Clinic HeidelbergHeidelberg, Germany; ^2^Department of Therapeutic Sciences, SRH University HeidelbergHeidelberg, Germany

**Keywords:** embodiment, affect, emotion, body feedback, embodied intersubjectivity, interaffectivity, psychopathology, embodied therapies

## Abstract

There is a growing body of research indicating that bodily sensation and behavior strongly influences one's emotional reaction toward certain situations or objects. On this background, a framework model of embodied affectivity^1^ is suggested: we regard emotions as resulting from the circular interaction between affective qualities or affordances in the environment and the subject's bodily resonance, be it in the form of sensations, postures, expressive movements or movement tendencies. Motion and emotion are thus intrinsically connected: one is *moved by movement* (perception; impression; affection^2^) and *moved to move* (action; expression; e-motion). Through its resonance, the body functions as a medium of emotional perception: it colors or charges self-experience and the environment with affective valences while it remains itself in the background of one's own awareness. This model is then applied to emotional social understanding or *interaffectivity* which is regarded as an intertwinement of two cycles of embodied affectivity, thus continuously modifying each partner's affective affordances and bodily resonance. We conclude with considerations of how embodied affectivity is altered in psychopathology and can be addressed in psychotherapy of the embodied self.

## Introduction

Emotions may be considered some of the most complex phenomena of subjective experience. This is mirrored by the host of different and often opposing emotion theories both in philosophy and psychology. Of the many attempts to reduce the complexity of emotions to a more simplified concept, two should be mentioned. The first focuses on their bodily component, as in the famous theory of James and Lange (James, 1884), simply put: we do not shiver because we are scared of the lion, but we shiver, and *this is* what we feel as our fear. In other words, emotions are feelings of bodily changes. This counter-intuitive assumption has been widely criticized for neglecting the intentional content or “aboutness” of emotions.

On the other hand, the contrary theory seems no less one-sided: according to prevailing cognitive approaches (Solomon, 1976; Lyons, 1980; Nussbaum, 2001), an emotion mainly consists in an act of evaluation or appraisal of a given situation. The bodily experience of emotions is then regarded as just an additional quale without further relevance (Gordon, 1987) or serving the limited purpose to assure us that an emotion is going on (Lyons, 1980). Again simplified: we believe or judge the lion to be dangerous, want to run away, and *this is* our fear of him. However, belief-desire concepts of emotions have been notoriously unable to capture their experiential and phenomenal aspect. A purely cognitive or functional approach to the phenomenon loses its peculiar self-affecting character. In particular, it fails to account for the changing *intensity* of emotions: it seems virtually impossible to indicate what a more intense anger, shame, or fear should be without referring to bodily experience (e.g., to one's increased sense of muscle tension, breath restriction, heated face or pounding heart). Cognitions as such do not differ in intensity. We may put the belief that “the lion is dangerous” into the comparative “the lion is very dangerous,” or we may repeat the thought with high frequency, but this does not yield a different affective experience unless we feel the “very” or the repetition as expressing a more activated, tense or stressful bodily state (Lang et al., 1993; Reisenzein, 1994). There is, however, no necessity and no indication to impose a linear causality model upon the complex phenomena of emotions (Boettinger, 2012). Given the divergent and inconclusive findings under the assumption of linear causality, models of circular causality may lead to a more appropriate understanding of emotional phenomena.

In the past decades a growing body of research on embodiment has demonstrated that not only bodily sensations, but also bodily postures, gestures and expressions are inherent components of emotional experience and tacitly influence the evaluation of persons, objects and situations as well as memory recall. To provide some examples:

- Riskind (1984) found that individuals recalled more negative life events when sitting in a slumped position, and more positive events when sitting in an upright position.
- Strack et al. (1988) demonstrated that activation of the smiling muscle (by asking participants to hold a pen between their teeth) caused participants to judge cartoons to be funnier than when smiling was inhibited by holding the pen between their lips.
- Cacioppo et al. (1993) reported that Chinese ideographs presented during arm flexion (an approach motion) were evaluated more positively than ideographs presented during arm extension (an avoidance motion; see also Neumann and Strack, 2000).
- Koch (2014; this issue) showed that an approach movement of the arms and a receptive movement of the hands caused a more positive attitude toward target objects than an avoidance movement; similarly, dynamic qualities of movement with smooth transitions caused more positive affect and a higher receptivity toward the environment than movement with sharp transitions.
- Cuddy et al. (2012) found that when people stood or sat for 7 min in a “power position” (different forms of extension of the body), they performed better in a subsequent mock job interview.
- Williams and Bargh (2008) showed that holding a hot cup of coffee elicits a “warmer” (more generous, caring) impression of a target person than holding a cup of iced coffee. Bodily felt warmth thus directly affected the interpersonal impression of warmth.
- Conversely, Zhong and Leonardelli (2008) found that people estimated the room temperature as being colder than before after they had experienced social exclusion from a group. Interpersonal coldness was thus felt as physical coldness. Correspondingly, Bargh and Shalev (2012) found that persons who experience social loneliness show an increased tendency to take warm baths or showers.
- The cleaning away of guilt is another interesting case: Meier et al. (2012) report a number of studies showing that cleansing can wash away feelings of guilt (Lee and Schwarz, 2011) or sin (Zhong and Liljenquist, 2006), and had a mildness influence on one's moral judgment (Schnall et al., 2008).
- Last but not least, Havas et al. (2010) found that the injection of botulinum toxin (Botox) into the frowning muscles impaired the understanding of negative semantic content such as criticism in a text which subjects had to read. This indicates that such understanding normally affords a slight frowning movement. On the other hand, injection of botulinum toxin into these muscles may significantly improve depressive symptoms in patients as has been shown in a randomized controlled trial by Wollmer et al. (2012). Obviously, negative evaluation of oneself as well as of semantic content is supported by corresponding facial expressions.

These and related research results may be summarized as follows:

1. When individuals adopt or produce emotion-specific postures, facial expressions or gestures, (a) they tend to experience the associated emotions, and (b) their behavior and also their preferences, judgement and attitudes toward objects or persons are thereby tacitly influenced.
2. Conversely, when individuals' expressive movements are *inhibited*, the experiencing of the associated emotions as well as the processing of corresponding emotional information is impaired. This is even the case when the information is presented in a merely cognitive or non-expressive way (as shown by the study of Havas et al., 2010, above).

Empirical findings thus show that embodiment has a far reaching influence on our emotional life. How may this influence be adequately understood? While we know that proprioceptive body feedback (based on afferent neural pathways from the body to the brain) is one of the responsible mechanisms (Hatfield et al., 1994; Koch, 2011), its interplay with the emotional perception and evaluation of a given situation still needs to be clarified. If we want to integrate the existing empirical research results into a comprehensive model of embodied affectivity, it seems advisable to follow a step-by-step approach: We will first consider emotions under different aspects, then we will try to integrate these aspects into an embodied and enactive concept of emotions. Finally, we will apply this concept to the special situation of social interactions or what may be called “embodied interaffectivity.”

## What are emotions?

In a first approximation, emotions may be regarded as affective responses to certain kinds of events of concern to a subject, implying conspicuous bodily changes and motivating a specific behavior (De Sousa, 2010). Accordingly, we will consider emotions under the aspects of (a) affective intentionality, (b) bodily resonance, (c) action tendency, and (d) function and significance.

*(a) Affective intentionality*. There is wide agreement among philosophers and psychologists that emotions are characterized by intentionality—they relate to persons, objects, events and situations in the world (see e.g., Solomon, 1976; Frijda, 1994; De Sousa, 2010). However, this intentionality is of a special kind: it is not neutral, but concerns what is particularly *valuable and relevant* for the subject. In a sense, emotions are ways of perceiving, namely attending to salient features of a situation, giving them a significance and weight they would not have without the emotion. Referring to Gibson's (1979) concept of affordances (that means, offerings in the environment that are available to animals, such as a tree being “climbable,” water “drinkable,” etc.), one could also speak of *affective affordances*: things appear to us as “important,” “worthwhile,” “attractive,” “repulsive,” “expressive,” and so on. Without emotions, the world would be without meaning or significance; nothing would attract or repel us and motivate us to act.

Of course, this meaning-making implies an evaluative or appraising component which should not, however, be conceived in terms of propositional attitudes (*believing that p is the case*, for example, believing that a lion is dangerous; cf. Lyons, 1980); otherwise, emotions could not be experienced by small children or higher animals lacking language. The evaluative aspect of affective intentionality is not dependent on verbally structured judgements, but on more basic cognitive-emotional schemes which are acquired in the course of affect-inducing experiences. Thus, an approaching lion will be immediately perceived *and* felt as a dangerous object once one has heard a lion's terrible roaring before, seen its leap toward a prey, etc. It has then acquired a threatening appearance which does not necessarily imply a belief such as “this is a lion,” “lions are dangerous,” etc. Of course there are emotional situations which are largely determined by higher forms of cognition (e.g., if an emotionally relevant information is provided in written form, or requires abstract concepts such as knowledge about an imminent stock market crash). But even then it is only the embodied response to the recognized situation that mediates its affective appeal and significance [see (b)]. Appraisal theories are highly relevant for explaining different emotional reactions of individuals on the basis of their preset attitudes, biases, beliefs, or judgements. But they are insufficient, when it comes to explain the holistic phenomenon of emotional experience itself^3^.

Moreover, the appraisal component may not be regarded as a mere cognitive judgement, because in emotions, *oneself is affected*. They always imply a particular relation to the feeling subject in its very core: through emotions, I experience *how it is for me* to be in this or that situation. *It is me* who is surprised, hurt, angry, joyful, etc. Affective intentionality is thus twofold: it discloses an affective or value quality of a given situation as well as the feeling person's own state in the face of it (Slaby and Stephan, 2008). To be afraid of an approaching lion (world-reference) means at the same time being afraid for oneself (self-reference). To feel envy toward another person means to begrudge her an advantage or success as well as to feel inferior and dissatisfied with oneself. Each emotion, thus, implies the two poles of feeling *something* and feeling *oneself* as inextricably bound together.

*(b) Bodily resonance*. How do we experience the affective qualities or affordances of a given situation? Emotions are experienced through what we call bodily resonance. This includes all kinds of local or general bodily sensations: feelings of warmth or coldness, tickling or shivering, pain, tension or relaxation, constriction or expansion, sinking, tumbling or lifting, etc. They correspond, on the one hand, to autonomic nervous activity (e.g., raised heartbeat, accelerated respiration, sweating, trembling, visceral reactions), on the other hand, to various muscular activations, bodily postures, movements and related kinaesthetic feelings (e.g., clenching one's fist or one's jaws, moving backwards or forwards, bending or straightening oneself, etc.). Particularly rich fields of bodily resonance are the face and the gut. Thus, for example, sadness may be felt locally as a lump in the throat, a tightening in the chest or in the belly, a tension around the eyes, a tendency to weep, or globally as a sagging tendency or a painful wave spreading through the entire body (Gendlin, 1967). Bodily resonance is also related to Damasio's concept of the “somatic markers,” consisting of interoceptive and proprioceptive feedback from the body that needs to be integrated with other more cognitive information in the frontal lobe of the brain in order to guide one's behavior, in particular in every day decision-making (Damasio, 1994, 1996).

In sum, as William James put it, the body is a most sensitive “sounding-board” in which every emotion reverberates (James, 1884), both within and between us. In addition, our bodies have a varying degree of permeability (“Durchlässigkeit”; Lewin, 1935), affectability and responsivity (e.g., Stern, 1985; Trevarthen, 2009) at any given point in time. The tired body is more permeable than the wake body, the drunk body more permeable than the sober body (Lewin, 1935). At the same time, these bodily feelings have an immediate repercussion on the emotion as a whole: Feeling one's heart pound in fear raises one's anxiety, feeling one's cheeks burn with shame increases the painful experience of exposure and humiliation (Ekman et al., 1972). Therefore, bodily feelings should not be conceived as a mere by-product or add-on, distinct from the emotion as such, but as the *very medium* of affective intentionality. Being afraid, for instance, is not possible without feeling a bodily tension or trembling, a beating of the heart or a shortness of breath, and a tendency to withdraw. It is *through* these sensations that we are anxiously directed toward a frightening situation.

According to traditional appraisal-theories (Lazarus, 1982), the evaluation of a given situation is a primary and separate component of emotions which precedes any bodily changes. From an embodied perspective, however, it is the lived body with its background sensations that is co-constitutive of the evaluation, which means that we should rather speak of an “embodied appraisal” (Prinz, 2004). For example, when feeling tired or exhausted, a familiar way uphill appears steeper and longer than normally. This appraisal does not result from a separate evaluative judgement, but from the very mismatch between one's bodily capacity and the task one faces. The hill is “too high,” that means it is perceived in this way *through* the tired, incapable body. Even in cases where emotionally relevant information is presented in merely abstract form (such as the text with negative content in the Botox study by Havas et al. see above), the evaluation obviously also depends on the simultaneous bodily resonance. More generally, our feeling body is the way we are emotionally related to the world, or in other words, affective experiences *are* bodily feelings-toward (Goldie, 2000). In emotions, there is no separation between an appraisal and a bodily component for they are only realized as a synthesis or “full circle” of all mutually interacting components.

*(c) Action tendency*. Bodily resonance of emotions is not restricted to autonomic nervous system activity or facial expression (which are in the focus of most empirical studies), but includes the whole body as being moved and moving. Fear, for example, does not only mean a raised heart beat or widely opened eyes but also the urge to break free, to flee or to hide (Sheets-Johnstone, 1999). The term “emotion” is derived from the Latin *emovere*, “to move out,” implying that inherent in emotions is a potential for movement, a directedness toward a certain goal (be it attractive or repulsive) and a tension between possible and actual movement. Correspondingly, Frijda (1986) has characterized emotions in terms of *action readiness*, according to the different patterns of action which they induce: approach (e.g., desire), avoidance (e.g., fear), being-with (enjoyment, confidence), attending (interest), rejecting (disgust), non-attending (indifference), agonistic (anger), interrupting (shock, surprise), dominating (arrogance), and submitting (humility, resignation).

Similarly, according to Kafka (1950) and De Rivera (1977), there exist four basic emotional movements: moving oneself “toward the other” (e.g., affection, mourning), moving the other “toward oneself” (e.g., desire, greed), moving the other “away from oneself” (e.g., disgust, anger) and moving oneself “away from the other” (e.g., fear, disgust). The four are related to the gestures of giving, getting, removing and escaping. These basic movements are connected to a bodily felt sense of expansion or contraction, relaxation or tension, openness or constriction, etc. In anger, for example, one feels a tendency of expansion toward an object in order to push it away from self. In affection, one feels a relaxation, opening and emanation toward an object or person. Emotions can thus be experienced as the directionality of one's potential movement, although this movement need not necessarily be realized in physical space; they are phenomena of lived space (Fuchs, 2007).

*(d) Functions and significance*. On the basis of the analysis so far, the role of emotions for the individual may be determined as follows: Emotions “befall us”; they interrupt the ongoing course of life in order to inform us, warn us, tell us what is important and what we have to react upon. They (re)structure the field of relevance and values; some of our plans, intentions or beliefs must be revised (Downing, 2000). Emotions thus provide a basic *orientation* about what really matters to us; they contribute to defining our goals and priorities. At the same time, they sketch out a certain scope and direction of possible responses, which are complementary to the *meaning* the emotion gives to the situation. Bodily resonance, autonomic arousal and musular activations make us become *ready to act*: in anger we prepare for attack, in fear we prepare for flight, in shame we want to hide or disappear, in love we want to approach and be approached. Emotion may thus be regarded as a bodily felt transformation of the subject's world, which solicits the lived body to action. However, even when the action tendency of emotions does not win through, they still retain an *expressive* function: by indicating the individual's state and possible action to others, they serve a communicative function in social life which will be explained in the section on “interaffectivity.”

## An embodied and extended concept of emotions

We now have gathered the necessary components that may be integrated into an embodied and extended model of emotions:

1. Emotions emerge as specific forms of a subject's bodily directedness toward the valences and affective affordances of a given situation^4^. They encompass subject and situation and therefore may not be localized in the interior of persons (be it their psyche or their brain). Rather, the affected subject is engaged with an environment that itself has affect-like qualities. For example, in shame, an embarrassing situation and the dismissive gazes of others are experienced as a painful bodily affection which is the way the subject *feels* the sudden devaluation in others' eyes. The emotion of shame is extended over the feeling person and his body as well as the situation as a whole (on this extended concept of affectivity cf. Schmitz et al., 2011).
2. Emotions imply two components of bodily resonance:
  - *a centripetal or affective component*, i.e., being affected, “moved” or “touched” by an event through various forms of bodily sensations (e.g., the blushing and “burning” of shame);
  - *a centrifugal or “emotive” component*, i.e., a bodily action readiness, implying specific tendencies of movement and directedness (e.g., hiding, avoiding the other's gaze, “sinking into the floor” from shame).

**Figure 1:**
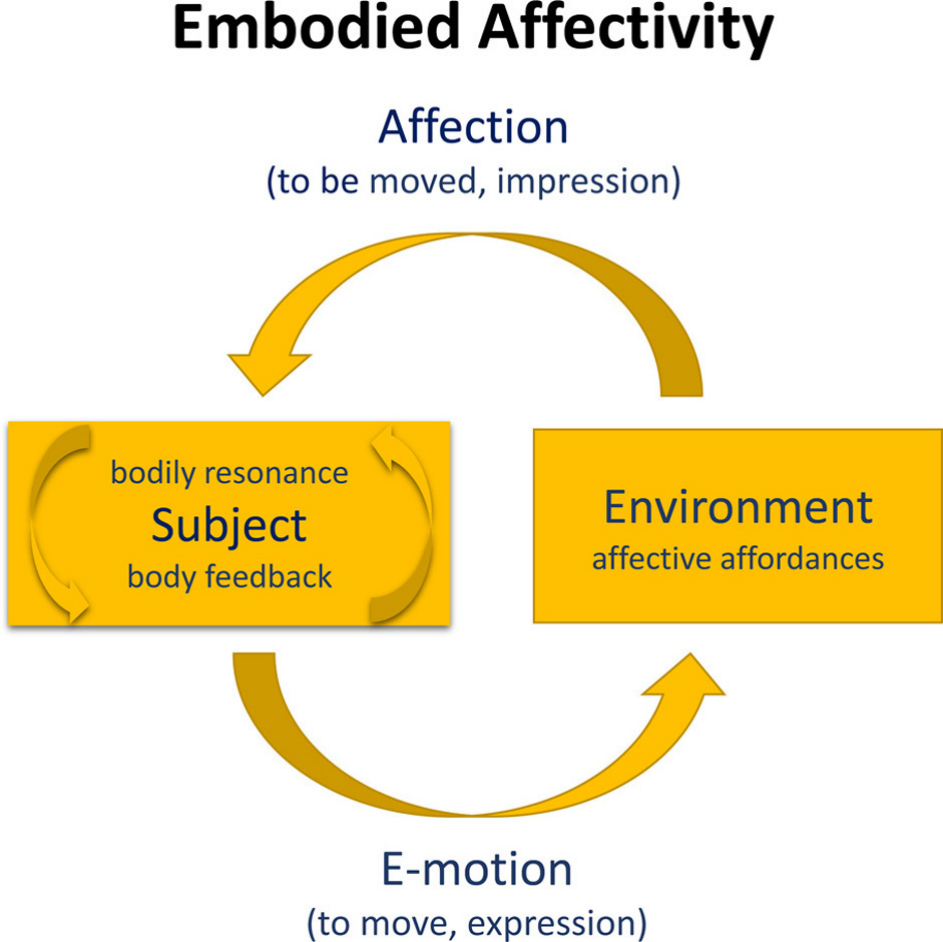
**Embodied affectivity**. We use phenomenological terminology in the model; in psychological terminology *subject* could be replaced by *person*, and *affection* by *affect*. Expression is constitutive of *e-motion* (centrifugal), impression is constitutive of *affection* (centripetal), resulting in interoceptive and proprioceptive body feedback or bodily resonance (arrows in *subject*). Emotion is mediated by the expressive ability (e.g., Laban, 1980), and affection is mediated by the permeability of the person system (Lewin, 1935). Both expression and impression (Wallbott, 1990) constitute the unity of movement and perception (cf. Weizsäcker, 1940), and form an integral part of our *personality*.

On this basis, feelings may be regarded as *circular interactions or feedback cycles* between centripetal affection and centrifugal e-motion (cf. Figure 1). Being affected by affective affordances of a situation triggers a specific bodily resonance (“affection”) which in turn influences the emotional perception and evaluation of the situation *and* implies a corresponding action readiness (“e-motion”). Affective intentionality consists in the entire interactive cycle, which is mediated by the resonance of the feeling body. Thus, in affectivity we are *moved by movement* (impression, affection) and *moved to move* (expression, e-motion), indicating the kinetic-kinaesthetic ambiguity of the body (Sheets-Johnstone, 1999).

3. Bodily resonance thus acts as the medium of our affective engagement in a given situation. It imbues, taints and permeates the perception of this situation without necessarily stepping into the foreground. In Polanyi's terms, bodily resonance is the *proximal*, and the perceived situation is the *distal*, component of affective intentionality, with the proximal component receding from awareness in favor of the distal (Polanyi, 1967). This may be compared to the sense of touch which is at the same time a self-feeling of the body (“proximal”) and a feeling of the touched surface (“distal”); or to the subliminal experience of thirst (“proximal”) which first becomes conspicuous as the perceptual salience of water flowing nearby (“distal”).
4. If the resonance or *affectability* of the body is modified in specific ways, this will change the person's affective perception accordingly. This is the common basis of the studies on embodiment and emotions that we mentioned above. Thus, a lack of resonance (e.g., after injection of botulinum toxin) will impede the perception of corresponding affective affordances in the environment. Conversely, increasing a certain bodily feeling (e.g., holding a hot cup of coffee), adopting a certain position or moving in a certain way favors the correlated affective perception. Thus, the different components of the affection-intention-motion cycle influence one another.

The last point is of particular psychotherapeutic importance, for it shows that emotions may not only be influenced by cognitive means (i.e., by changing the cognitive component of the cycle), but also by modifying the bodily resonance. It can be diminished as well as increased. The first is the case in habitual *body defences*: When an emotion emerges, one often tends to defend against it by bodily counteraction: suppressing one's tears or cries, compressing one's lips, tightening one's muscles, keeping a stiff posture, “pulling oneself together,” etc. This often happens unconsciously, as part of one's early acquired bodily *habitus* (cf. Bourdieu, 1990). On the other hand, the experience of vague or diffuse emotions may be enhanced and differentiated by carefully attending to the bodily feelings and kinaesthetic tendencies which these emotions imply, in order to render them accessible to verbal explication in psychotherapy.

In concluding the section, we may add that the connections of affectivity and embodiment that we have presented in a general model show considerable cultural and individual variations. The culture-specific forms of emotional expression or restraint as well as the habitus of a person which has incorporated basic attitudes such as introversion or extroversion, shyness or pride, submissiveness or dominance, etc., have become part of the individual body memory (Fuchs, 2012) and thus influence the circular relations between affective affordances, bodily resonance and emotional response in a given situation. Here lies a rich field for future research into the impact of culture and biography on the embodiment of emotions.

## Interaffectivity

As we have seen, emotions imply embodied action tendencies. More specifically, in the social sphere they are characterized by various potential movements toward, or away from, an actual or implicit *other* (Kafka, 1950; De Rivera, 1977), i.e., they are essentially *relational*. As such, they are not only felt from the inside, but also displayed and visible in expression and behavior, often as bodily tokens or rudiments of action^5^. The facial, gestural and postural expression of a feeling is part of the bodily resonance that feeds back into the feeling itself, but also induces processes of *interaffectivity:* Our body is affected by the other's expression, and we experience the kinetics and intensity of his emotions through our own bodily kinaesthesia and sensation.

Emotions thus imply two components of bodily resonance or feedback:

- Self- or individual resonance: proprio- and interoceptive feedback providing the organism with useful information from body postures, gestures or sensations (Zajonc and Markus, 1984; Hatfield et al., 1994: the body as “interface” between cognition and affect).
- Interactional or interbodily resonance: dynamic mutual feedback between two bodies (e.g., you lift your arms and I feel slightly “uplifted”). This body feedback can occur through the visual, auditory or tactile channel (such as from a handshake or an embrace; Koch, unpublished Manuscript), but also through the kinaesthetic channel (such as from directional movements; e.g., Koch et al., 2011).

This means that in every social encounter, two cycles of embodied affectivity (cf. Figure 1 above) become intertwined, thus continuously modifying each subject's affective affordances and resonance. This complex process may be regarded as the bodily basis of empathy and social understanding.

**Figure 2:**
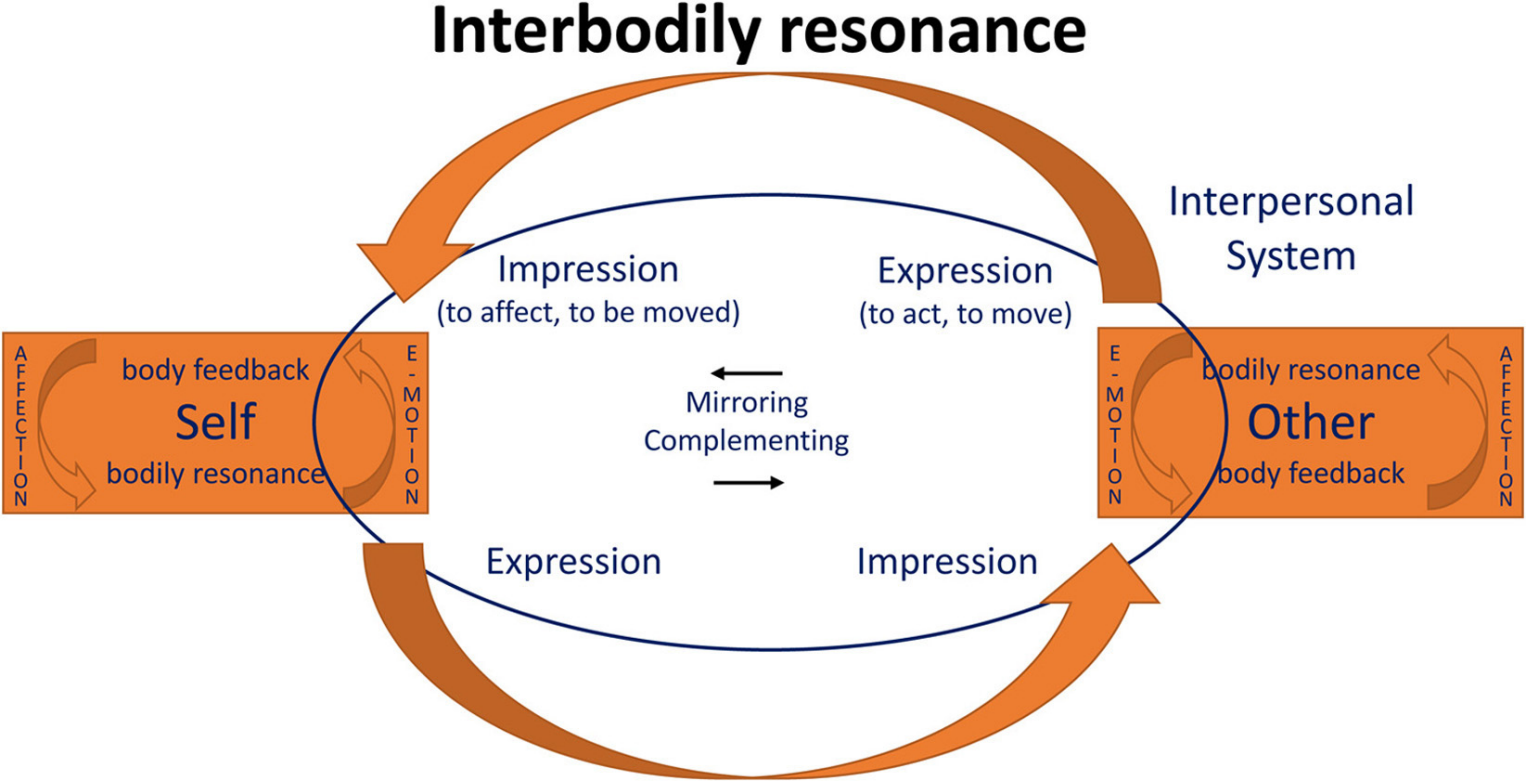
**Interaffectivity**. The figure is an integration of Koch (2011), Froese and Fuchs (2012), and Fuchs (2013). Interaffectivity includes body feedback (i.e., the impression function within self and other) which is necessary for interbodily resonance. Components of interbodily resonance are: mirroring or complementing movements, body awareness (via proprioceptive body feedback), and kinaesthetic empathy; they are psychotherapeutically important in phenomena such as somatic countertransference (Pallaro, 2002).

To illustrate this (Figure 2), let us assume that the SELF (A) is a person whose emotion, e.g., anger, manifests itself in typical bodily (facial, gestural, interoceptive, etc.) changes. He feels the anger as the tension in his face, the sharpness of his voice, the arousal in his body etc. This resonance is an *expression* of the emotion at the same time, i.e., the anger becomes visible and is perceived as such by the OTHER (B). But what is more, the expression will also produce an *impression*, namely by triggering corresponding or complementary bodily feelings in the OTHER. Thus, A's sinister gaze, the sharpness of his voice or expansive bodily movements might induce in B an unpleasant tension or even a jerk, a tendency to withdraw, etc. (similarly, shame that one witnesses may induce embarrassed aversion, sadness a tendency to connect and console, and so forth). Thus, B not only sees the emotions in the A's face and gesture, but also senses it with his own body, through his own bodily resonance.

However, it does not stay like this, for the impression and bodily reaction caused in B in turn becomes an expression for A. It will immediately affect his bodily reaction, change his own expression, however slightly (e.g., increasing or decreasing his expression of anger), and so forth. This creates a circular interplay of expressions and reactions running in split seconds and constantly modifying each partner's bodily state. They have become parts of a dynamic sensorimotor and interaffective system that connects both bodies in *interbodily resonance* or *intercorporality* (Merleau-Ponty, 1964). Of course, the signals and reactions involved proceed far too quickly to become conscious as such. Instead, both partners will experience a specific feeling of being connected with the other in a way that may be termed “mutual incorporation” (Fuchs and De Jaegher, 2009). Each lived and felt body reaches out, as it were, to be extended by the other. In both partners, their own bodily resonance mediates the perception of the other. It is in this sense that we can refer to the experience of the other in terms of an embodied perception, which, through the interaction process, is at the same time an embodied communication.

No mental representation is necessary for this process. There is no strict separation between the inner and the outer, as if a hidden mental state in X produced certain external signs, which Y would have to decipher. For X's anger may not be separated from its bodily expression; and similarly, Y does not perceive X's body as a mere object, but as a living, animate and expressive body that she/he is coupled with.

Nor is a simulation required for the process of mutual incorporation. We certainly do not simulate the other's angry gaze or voice, even less his anger, but rather feel tense, threatened or even invaded by his expressive bodily behavior. Bodily sensations, tensions, action tendencies, etc. that arise in the interaction do not serve as a separate simulation of the other person, but are fed into the mutual perception. In Polanyi's terms, one could also say that the felt bodily resonance is the *proximal*, the other's perceived body is the *distal* component of one's empathic perception, with the proximal component receding from awareness in favor of the distal (Polanyi, 1967). Stuart (2012) has recently coined the term “enkinesthesia,” that means, “feeling one's own movements into the other,” or: empathy through subliminal co-movement. It is in this sense that we can refer to the experience of the other in terms of “embodied” perception, which, through the interaction process, is at the same time an “embodied” communication. In Merleau-Ponty's account:

“The communication or comprehension of gestures comes about through the reciprocity of my intentions and the gestures of others, of my gestures and the intentions discernible in the conduct of other people. It is as if the other person's intentions inhabited my body and mine his” (Merleau-Ponty, 1962).

As we can see, the concept of mutual incorporation leads to the opposite of the representationalist account: Primary social understanding is not an inner modeling in a detached observer, but the other's body extends onto my own, and my own extends onto the other.

This can perhaps best be studied in early childhood. Emotions primarily emerge from and are embedded in dyadic interactions of infant and caregiver. Stern (1985) has shown in detail how emotions are cross-modally expressed, shared, and regulated. Infants and adults experience joint affective states in terms of dynamic flow patterns, intensities, shapes, and vitality affects (for example, *crescendo* or *decrescendo*, fading, bursting, pulsing, effortful or easy, etc.) in just the way that music is experienced as affective dynamics. This includes the tendency to mimic and synchronize each other's facial expressions, vocalizations, postures, movements, and thus to converge emotionally (Condon, 1979; Hatfield et al., 1994). All this may be summarized by the terms *affect attunement* and *interaffectivity* (Stern, 1985; p. 132): The emerging affect during a joyful playing situation between mother and infant may not be divided and distributed among them. It arises from the “in-between,” or from the over-arching process in which both are immersed. *Affect attunement* is carried by *kinaesthetic empathy* (Kestenberg, 1975; Fischman, 2008), which is also employed in dance/movement therapy diagnostics and intervention (for a systematization of forms of attunement and mirroring see Eberhard-Kaechele, 2012).

**Figure 3:**
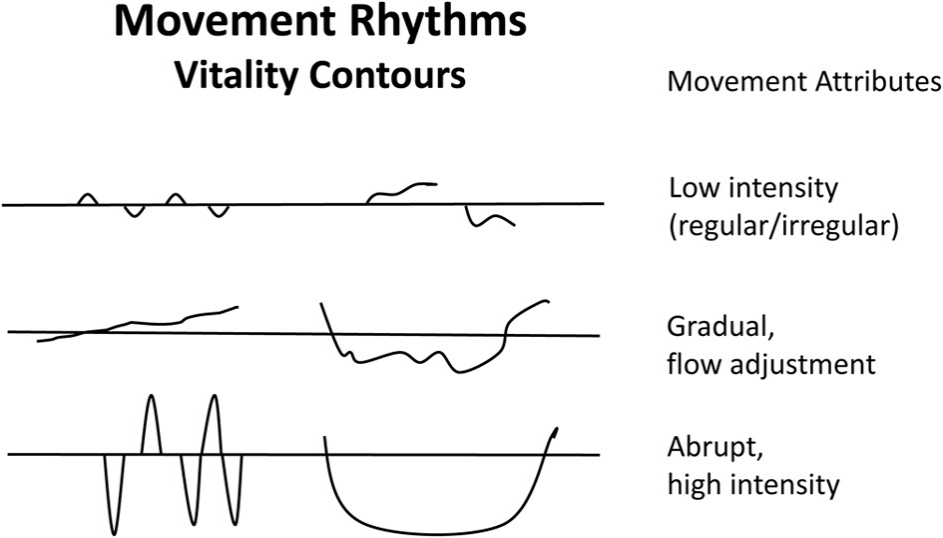
**Movement rhythms**. Example of rhythm writing to capture affect expression and vitality contours (Kestenberg and Sossin, 1973, 1979; Stern, 2010).

Affect attunement was first investigated by Kestenberg (1975); Kestenberg and Sossin (1973, 1979), who systematized it into quality and shape attunement and described developmental regularities and sequences. Kestenberg emphasized that in the individuation process, partial attunement of mother and child was more productive than complete attunement to serve the child's development. A basic dimension of meaning are smooth vs. sharp reversals between rhythms (Koch, 2011). Via kinaesthetic empathy, researchers can notate body rhythms (Figure 3) that may be used to analyse affect attunement differentially (Koch, 2014). These rhythm curves reflect what Stern calls “vitality affects” or “vitality contours” (Stern, 1985, 2010). Shared vitality affects then form a vital part of our emotions.

Thus, emotions are not inner states that we experience only individually or that we have to decode in others, but primarily *shared states* that we experience through interbodily affection. Even if one's emotions become increasingly independent from another's presence in the course of childhood, intercorporality remains the basis of empathy: There is a bodily link which allows emotions to immediately affect the other and thus enables empathic understanding without requiring a Theory of Mind or verbal articulation (Fuchs and De Jaegher, 2009). On this basis, we have created a short scale that measures the degree of feeling understood by and understanding of others through movement. The *Embodied Intersubjectivity Scale* (*EIS*; see Appendix) consists of ten items measuring the degree of closeness created by different forms of attuning and mirroring in movement. It complements the *Body Self-Efficacy Scale* (*BSE;* see Appendix), which measures the body-based “I can't” of a person (Husserl, 1952) also with 10 items. Perceived body self-efficacy is related to a positive body image, positive movement-based affect (MBAS; Koch, 2014) and the ability for embodied interaffectivity (Appendix).

## (Psycho)pathological implications

The model of embodied affectivity that we have presented may gain additional plausibility from different kinds of disturbances which occur in psychopathology. We will illustrate its implications by using the examples of (1) anxiety disorder, (2) depression, (3) Parkinson's disease, (4) alexithymia, and (5) autism.

1. *Anxiety disorders* are characterized by a heightened alert of the body which reacts to threatening affective affordances in the environment with intense feelings of oppression mainly in throat, breast or gut (corresponding physiologically to muscular tension, trembling, palpitation, hyperventilation, sweating, etc.)^6^. This bodily affection motivates, on the one hand, a hypervigilant perception: The anxious person scans the environment for threatening cues and anticipates lurking danger. On the other hand, the bodily resonance also implies a specific action tendency, namely to escape the oppressing situation through flight or to avoid it in advance. Phobias particularly related to space, such as agoraphobia, claustrophobia or acrophobia, dynamize the otherwise static quality of experienced space and illustrate the overall spatial structure of anxiety as encompassing body and environment.
2. In contrast, a lack or loss of bodily affectability is characteristic of severe *depression*. The constriction, rigidity and missing tension-flow modulation (neutral flow; Kestenberg, 1975) of the lived body in depression leads to a general emotional numbness and finally to affective depersonalization (Fuchs, 2005). The deeper the depression, the more the affective qualities and atmospheres of the environment fade. The patients are no longer capable of being moved and affected by things, situations or other persons. They complain of a painful indifference, a “feeling of not feeling” and of not being able to sympathize with their relatives any more. In his autobiographical account, Solomon describes his depression as “… a loss of feeling, a numbness, (which) had infected all my human relations. I didn't care about love; about my work; about family; about friends … ” (Solomon, 2001; p. 45). Thus patients feel disconnected from the world; they lose their participation in the interaffective space that we normally share with others (Fuchs, 2013).
3. In some way similar to depression, we find in progressed *Parkinson's disease* a “freezing” of face and body, which leads to loss of emotional expressivity. As a result, patients tend to experience a decreased intensity of their emotions and complain of no longer being able to participate in interaffective exchange with others as before. Studies have also found that patients with Parkinson's disease were less accurate than healthy controls in decoding angry, sad and disgusted facial expressions of others, pointing to a lack of bodily resonance as the proximal component of affective perception (see Mermillod et al., 2011, for an overview).
4. Persons characterized by *alexithymia* have marked difficulties to identify, differentiate and describe their own emotions, while at the same time being unable to recognize the affective nature of bodily sensations associated with certain emotions (Taylor and Taylor, 1997). This is often accompanied by a lack of understanding of the feelings of others, which leads to unempathic emotional responding (Hesse and Floyd, 2008). Alexithymia is particularly frequent in patients with *somatoform disorders* who are have often problems to relate their bodily resonance to corresponding affective situations, leading to detached feelings of pressure, burning, pain, etc., which are then attributed to assumed somatic illnesses (Duddu et al., 2003). Moreover, interoceptive sensitivity, measured as a person's ability to accurately perceive one's heartbeats at rest, has been found to be reduced in somatoform patients which was associated with a reduced capacity of emotional self-regulation (Pollatos et al., 2011; Weiß et al., 2014). Interoceptive sensitivity normally facilitates successful self-regulation by providing a fine-tuned feedback of the present emotional state (Füstös et al., 2011).
  What is obviously lacking in alexithymia is the proximal-distal structure of affective intentionality: Whereas bodily resonance normally functions as the proximal medium of our affective perception, for alexithymic patients their bodily reactions seem unrelated to affective affordances of a given situation, which means that the full circle of affectivity does not come about. Bodily sensations of resonance either are not felt at all, or they may come to the fore separately, instead of receding from awareness in favor of affective intentionality. In both cases, this is connected to a sense of emotional detachment of patients from themselves. Pathogenetically, a lack of interaffective mirroring and feedback in early childhood seems to play a major role: If caregivers are incapable of recognizing and validating emotional expressions in the child, this can impair the child's capacity to understand and differentiate emotional states within himself as well as in others (Graerne and Bagby, 2000).
5. Finally, *autism* or *autistic spectrum disorder* may be regarded as a disturbance of embodied interaffectivity, namely as a lack of perceiving others' expressions, gestures and voicings in terms of affective affordances. Correspondingly, eye tracking studies have shown that children with autism focus on inanimate and irrelevant details of interactive situations while missing the relevant social cues, e.g., neglecting the eyes and mouths of protagonists (Klin et al., 2002). Another study asked children to sort people who varied in terms of age, sex, facial expressions of emotion and the hat that they were wearing (Weeks and Hobson, 1987). In contrast to typical children who grouped pictures by emotional expressions, the participants with autism grouped the people by the type of hat they were wearing. Generally, they prefer to attend to inanimate objects over other humans (Klin et al., 2003; Jones et al., 2008). Furthermore, while imitation and co-movement serves as a major instrument for early affect attunement and social cognition, several studies have found that autistic children do not readily imitate the actions of others (Smith and Bryson, 1994; Hobson and Lee, 1999).
  As a result of these deficiencies, there is a general lack of the embodied or kinaesthetic empathy that normally mediates the affective perception of the other. The feedback cycles of mutual incorporation are not achieved; instead, for children with autism the others remain rather mysterious, detached objects whose behavior is troublesome to predict. According to embodied and enactive approaches, what these children primarily lack is not a theoretical concept of others' minds (Klin et al., 2003; Gallagher, 2004; De Jaegher, 2013). This is supported by the fact many autistic symptoms such as lack of emotional contact, anxiety or agitation are already present in the first years of life, i.e., long before the supposed age of 4–5 years to acquire a Theory of Mind. Much rather, high-functioning autistic persons often develop precisely an explicit “Theory of Mind” approach to emotions, i.e., they learn to infer or “figure out” what emotion the other is experiencing (Grandin, 1995).

## Embodied therapies

Our model of embodied affectivity can be elucidating for the interpersonal processes taking place on a non-verbal level in psychotherapy and for the explicit thematization of bodily experience in body psychotherapy and dance movement therapy. These approaches use non-verbal modalities to start change processes, to gain access to affect and memories that dominated in a former situation—actualizations that are important, for example, in trauma treatment (Caldwell, 2012; Eberhard-Kaechele, 2012). Embodied therapies are increasingly framed in non-linear causality, enactive, ecological and dynamic systems approaches (cf. Koch and Fishman, 2011), to account for the complexity of motor processes and their interwovenness with brain functions and sociocultural/environmental factors.

The embodied affectivity model allows us to locate disorders on the continuum of e-motion and affectivity and to plan embodied interventions accordingly. *Anxiety* (1) for example, can be addressed and be alleviated by engagement in low intensity and gradual swaying movements—particularly with advancing movement in the horizontal plane—which are part of many meditative circle dances (Koch, 2011), strengthening their ability to calm down and perceive their environment as less threatening. *Depression* (2) can be temporarily alleviated by moving into high arousal, high intensity, abrupt movements with round reversals (such as in jogging or dancing) particularly in the vertical plane (Koch et al., 2007) which awakens joy and vitality, and decreases negative affect. Persons affected from *Parkinson* (3) profit from Tango Argentino (Duncan and Earhart, 2012),—characterized by its mostly low intensity abrupt movements and turns with flow adjustment, which address initiation, balance and gait, but also intersubjective sensitivity,—and from expressive dance training, which strengthens their expressive abilities. *Alexithymia* (4) is common in both somatoform and autistic populations. *Somatoform patients* benefit from structured authentic movement interventions (*The Body Mind Approach (TBMA)*, Payne and Stott, 2010) including a partner exchange, which support the connections between feeling and verbalization; and *autists* (5) from mirroring in movement—including structured authentic movement—, which can improve their intersubjective abilities (Koch et al., 2014b). This mostly evidence-based literature on the effects of movement therapy on (psycho-)pathological conditions has been summarized in Koch et al. (2014a).

Dance movement therapy starts on the moving and e-motion side (e.g., Levy, 2005), whereas embodied therapies such as focusing (Gendlin, 1967) and functional relaxation (Fuchs, 1997) start on the sensing and affectivity side of the model. Most experienced body psychotherapists—no matter which background they work from—integrate both sides in a balance of sensing and moving (e.g., Lahmann et al., 2010; Caldwell, 2012). A focus on breathing can help find the basis of this balance (e.g., Williams et al., 2007). Rogers (1951) already pointed out that persons entering into a sensing, reflective, and affective mode during the process of therapy, pausing and giving room to integrate the bodily feedback into the progression of a therapeutic session, are the ones that profit most from psychotherapy. Damasio (1994, 1996), in his somatic marker hypothesis, specified that no decision of practical relevance can produce authentic results without interoceptive and proprioceptive feedback from the body. Embodied therapies can help the individual to access this somatic information and to take it into account for daily living—a step that becomes increasingly difficult for many persons in Western societies with their largely exteroceptive focus.

## Conclusion

In sum, emotions result from the body's own feedback and the circular interaction between affective affordances in the environment and the subject's bodily resonance, be it in the form of sensations, postures, expressive movements, or movement tendencies. Through its resonance, the body functions as a *medium* of emotional perception.

Our account places particular emphasis on the intersubjective dimension of affectivity. In interaffectivity, our body is tacitly affected by the other's expression, and we experience the kinetics and intensity of his emotions through our own bodily kinaesthesia and sensation. This means that in every social encounter, two cycles of embodied affectivity become intertwined, thus continuously modifying each partner's affective affordances and resonance. Infant research demonstrates how the mutual bodily resonance of facial, gestural and vocal expression engenders our primary affective attunement to others. From birth on, the body is embedded in intercorporality, and thus becomes the medium of interaffectivity. Hence, affects are not enclosed in an inner mental sphere to be deciphered from outside, but come into existence, change and circulate between self and other in the interbodily dialog. Emotions are neither individual nor unidirectional phenomena; they operate in cycles that can involve multiple people in processes of mutual influence and bonding. These processes of embodied interaffectivity as well as their disturbances are of major importance for psychiatry, psychosomatics, and psychotherapeutic interactions and can be addressed in embodied therapies.

### Conflict of interest statement

The authors declare that the research was conducted in the absence of any commercial or financial relationships that could be construed as a potential conflict of interest.

## Footnotes

^3^The focus of this article is on emotions. The various interfaces with cognition result naturally from the intrinsic connection of emotion and cognition (e.g., Zajonc and Markus, 1984); to address them in the article would exceed its scope and length. It would in fact not even be necessary, since each situation that affects us emotionally does also concern us cognitively, and vice versa. I have to recognize the lion in order to feel afraid of it; I have to understand the words that insult me cognitively before they can create an affective affordance for me. Just as there is no cognition without emotion, at least in the sense of emotionally driven attention and interest, there is also no emotion without a cognitive grasp of the given situation.

^4^As pointed out above, the concept of affective intentionality and affective affordances also implies components of cognition (schemes, attitudes, beliefs, etc.) which shape the individual's emotional perception of, and response to a situation. Perceiving a goal in a football match, for example, will elicit quite different emotional responses depending on which team one supports. However, the focus of our model lies not on such preset attitudes, beliefs, biases etc. but on explaining the phenomenon of emotional experience as a whole (for which the term “embodied appraisal” (Prinz, 2004) seems much more adequate than the predominantly discussed “emotional appraisal”).

^5^According to (Darwin, 1872/1904), emotional expressions once served particular action functions (e.g., baring one's teeth in anger to prepare for attack), but now accompany emotions in rudimentary ways in order to communicate these emotions to others. Evolutionary psychologists have advanced the hypothesis that hominids have evolved both with increasingly differentiated facial expressions and with sophisticated capabilities of understanding these affect displays. In any case, though strongly varying between and within cultures, emotional expression is a crucial facet of interpersonal communication in all societies.

^6^Similarly, in Posttraumatic Stress Disorder (PTSD) there is an increased bodily responsivity (racing heart, dyspnea, fear-sweat, sickness) to certain environmental triggers that are related to former traumatic experiences (sights, sounds, smells, etc.), that means, the body resonance is shaped by a traumatic body memory (van der Kolk, 1994; Fuchs, 2012).

## Appendix

### Body self-efficacy-scale (BSE; Koch, unpublished manuscript)

Please answer how the following statements apply to you on a scale from 0 to 5 with 0 representing “applies not at all” and 5 representing “applies exactly”:

**Table:**

1. I can move well.	0	1	2	3	4	5
2. My movements are beautiful.	0	1	2	3	4	5
3. My body is flexible.	0	1	2	3	4	5
4. I have many bodily constraints.	0	1	2	3	4	5
5. My body is lifeless and inert/numb.	0	1	2	3	4	5
6. I can easily jump over an obstacle of medium size.	0	1	2	3	4	5
7. My body feels like “in pieces.”	0	1	2	3	4	5
8. My body often feels like it does not belong to me.	0	1	2	3	4	5
9. I can move elegantly/with grace.	0	1	2	3	4	5
10. I can express myself in movement.	0	1	2	3	4	5

*Internal Consistency *BSE*: *Cronbach's alpha* = 0.75 (students; *n*=63) and 0.83 (patients; *n* = 83) on the German version*.

### Embodied intersubjectivity scale (EIS; Koch, unpublished manuscript)

Please think about the last situation in which you have moved with others in a group (e.g., in movement therapy, in dancing). In how far did the following statements apply to you on a scale from 0 to 5 with 0 representing “applies not at all” and 5 representing “applies exactly”:

**Table:**

1. I can pick up the movements of others.	0	1	2	3	4	5
2. Through movement I can transmit/communicate aspects of myself.	0	1	2	3	4	5
3. Through the movement of others, I realize how they feel (e.g., joy, tension).	0	1	2	3	4	5
4. I can accompany others in movement (“mirror” movement).	0	1	2	3	4	5
5. I can recognize how others feel through joint movement.	0	1	2	3	4	5
6. Through joint movement a connectedness arises.	0	1	2	3	4	5
7. If others move in synch with me, I feel accepted by them.	0	1	2	3	4	5
8. Something new can emerge in moving with others.	0	1	2	3	4	5
9. I can understand, what others want to express with movement.	0	1	2	3	4	5
10. If another person moves in synch with me I feel understood.	0	1	2	3	4	5

*Internal Consistency *EIS*: *Cronbach's alpha* = 0.87 (students; *n* = 63) and 0.90 (patients; *n*=83) on the German version*.

Both measures had been pretested in a longer version on a sample of 80 psychology students at the University of Heidelberg and had been cut down from an item pool of twice the amount of items using the criterion of internal consistency scores. Both, the BSE and the EIS, were then tested with a sample of 63 students of therapy sciences at SRH University of Heidelberg, resulting in a *Cronbach's alpha* (*BSE*) of 0.75; and a *Cronbach's alpha* (*EIS*) of 0.87 (Kelbel, unpublished thesis). They were further employed in the context an RCT on movement therapy with schizophrenic and autistic populations (*n* = 83; 42 schizophrenic patients and 41 Autism Spectrum Disorder, mostly high functioning) and were found reliable for these patient groups (*Cronbach's alpha BSE* = 0.83; *Cronbach's alpha EIS* = 0.90; Kelbel, unpublished thesis).

The BSE (of both student and patient sample data) was validated with the Ryckman Scale (Ryckman et al., 1982) a standardized questionnaire on perceived physical ability. The two scales showed a correlation of *r* = 0.55, *p* < 0.01, indicating high agreement, even though, Ryckman et al. did not cover the aesthetic aspects included in the BSE.
